# Regulatory Functions of Cellular Energy Sensor SNF1-Related Kinase1 for Leaf Senescence Delay through ETHYLENE- INSENSITIVE3 Repression

**DOI:** 10.1038/s41598-017-03506-1

**Published:** 2017-06-09

**Authors:** Geun-Don Kim, Young-Hee Cho, Sang-Dong Yoo

**Affiliations:** Department of Life Science, College of Life Science and Biotechnology, KOREA University, 02841 Seoul, Korea

## Abstract

Aging of living organisms is governed by intrinsic developmental programs, of which progression is often under the regulation of their cellular energy status. For example, calorie restriction is known to slow down aging of heterotrophic organisms from yeasts to mammals. In autotrophic plants cellular energy deprivation by perturbation of photosynthesis or sugar metabolism is also shown to induce senescence delay. However, the underlying molecular and biochemical mechanisms remain elusive. Our plant cell-based functional and biochemical assays have demonstrated that SNF1-RELATED KINASE1 (SnRK1) directly interacts, phosphorylates, and destabilizes the key transcription factor ETHYLENE INSENSITIVE3 (EIN3) in senescence-promoting hormone ethylene signaling. Combining chemical manipulation and genetic validation using extended loss-of-function mutants and gain-of-function transgenic lines, we further revealed that a SnRK1 elicitor, 3-(3,4-dichlorophenyl)-1,1-dimethylurea enables to slow down senescence-associated leaf degreening through the regulation of EIN3 in *Arabidopsis*. Our findings enlighten that an evolutionary conserved cellular energy sensor SnRK1 plays a role in fine-tuning of organ senescence progression to avoid sudden death during the last step of leaf growth and development.

## Introduction

Senescence is a natural process of aging and preludes to death. In heterotrophic microbes and animals calorie restriction is known to delay aging and thus extends lifespan^1, 2^. The genetic perturbation of glucose metabolic processes delays vegetative organ senescence in *Arabidopsis* as shown in a glucose sensor HEXOKINASE1 deficient mutant *gin2-1* ^3^. Leaf senescence is also delayed in whole plants under darkness, in which condition cellular energy level is lowered, although individual dark-treated leaf initiates senescence^4^. These suggest that calorie restriction/cellular energy deprivation–inducible delay of organ senescence is an evolutionarily conserved developmental physiology in autotrophic plants as well.

Plant vegetative organ senescence often accompanies reproductive and/or dormant meristematic organ formation and development^5^. Upon senescence initiation, subcellular organelles and their biochemical constituents are disassembled, degraded, and remobilized in vegetative organs to support the growth of newly emerging shoots and roots and the development of fruits and seeds^6, 7^. Therefore, vegetative organ senescence plays an important role in reproductive success and eventually the perpetuation of plant species. Even so, because of the destructive nature of senescence processes, its initiation and progression are under the precise control of coordinated internal and external signaling cues^8, 9^.

Light drives photosynthesis and severs as a natural external signal, and thereby conveys pivotal information regulating plant growth as well as senescence. Detached leaves senesce in the absence of light and its progression is generally accelerated or decelerated depending on phytohormones engaged internally^9, 10^. As a small molecule product of plant cells, ethylene has long been studied for its promoting effects on plant organ senescence^11^. And hence, the management of ethylene production and accumulation has been an imperative postharvest practice for vegetable crops and fruits with regards to their shelf life extension^12^.

In a model plant *Arabidopsis* ethylene signaling pathway encompasses ETHYLENE RESPONSE1 (ETR1) and four other redundant receptors that transduce the hormone signal to ETHYLENE INSENTIVIE2 (EIN2), and then EIN3 and EIN3-LIKE1 (EIL1) in the nucleus^13, 14^. These key transcription factors in ethylene signaling activate specific target gene expression and their gene products involve in changes of cellular physiology. Beyond a canonical ethylene signaling pathway, EIN2/ORESARA2/3 (ORE2/3) dependent modulation of *miRNA164* provides a trifurcated signaling pathway to regulate the expression of a central transcription factor in senescence-related gene expression program, *NAC2/ANAC092/ORE1* (refs 15–17), gene products of which induce late senescence-associated gene expression including *SENESCENCE-ASSOCIATED GENE12* (*SAG12*)^18^^,^. Even though ethylene is not a senescence initiation factor^19^, ethylene-EIN3 signaling plays a key role in the regulation of senescence progression, thus any functional perturbation of EIN3 is apt to regulate plant organ senescence^20^.

Intracellular sugar starvation/energy deprivation signaling that is known to delay organismal senescence activates evolutionarily conserved Ser/Thr protein kinase SUCROSE-NON-FERMENTATION1 (Snf1) in yeasts, AMP-ACTIVATED PROTEIN KINASE (AMPK) in mammals, and Snf1-RELATED KINASE1 (SnRK1) in plants. Two α–subunit isoforms of SnRK1 complexes present in *Arabidopsis* genome: *Arabidopsis* Snf1 KINASE HOMOLOG 10 (AKIN10) and AKIN11 (refs 21 and 22). AKIN10 is activated by 3-(3,4-dichlorophenyl)-1,1-dimethylurea (DCMU)^21^, which blocks photosystem II (PSII) electron transport chain leading to calorie restriction^23^. Its activation is also triggered by hypoxia leading to suppression of mitochondrial aerobic respiration resulting in cellular energy deprivation^21, 24, 25^.

AKIN10 activation switches catabolic pathways on and anabolic pathways off, which brings about energy homeostasis and metabolic adaptation leading to cell viability enhancement and organ senescence delay^21, 22, 24^. For example, simple over-expression of AKIN10 that mimics cellular energy deprivation to some extend by harboring phosphorylated AKIN10 enables to delay natural leaf senescence^21, 24^. Even so, exact targets and detailed pathways underlying AKIN10 activity involved in the plant organ senescence delay remain less well understood.

In this study, we revealed that AKIN10 directly interacts, phosphorylates and antagonistically modulates EIN3, and thereby delays ethylene-promoted plant organ senescence. Consistently a chemical elicitor of AKIN10 activity DCMU that is frequently used to study energy flow in photosynthesis slows down senescence progression through destabilization of EIN3 in *Arabidopsis*. Our findings pinpoint a regulatory mechanism of an evolutionarily conserved cellular energy sensor AKIN10 in modulating of plant organ senescence progression to avoid sudden death of plant cells during senescence.

## Results

### SnRK1/AKIN10 directly interacts with and phosphorylates EIN3

**Figure 1:**
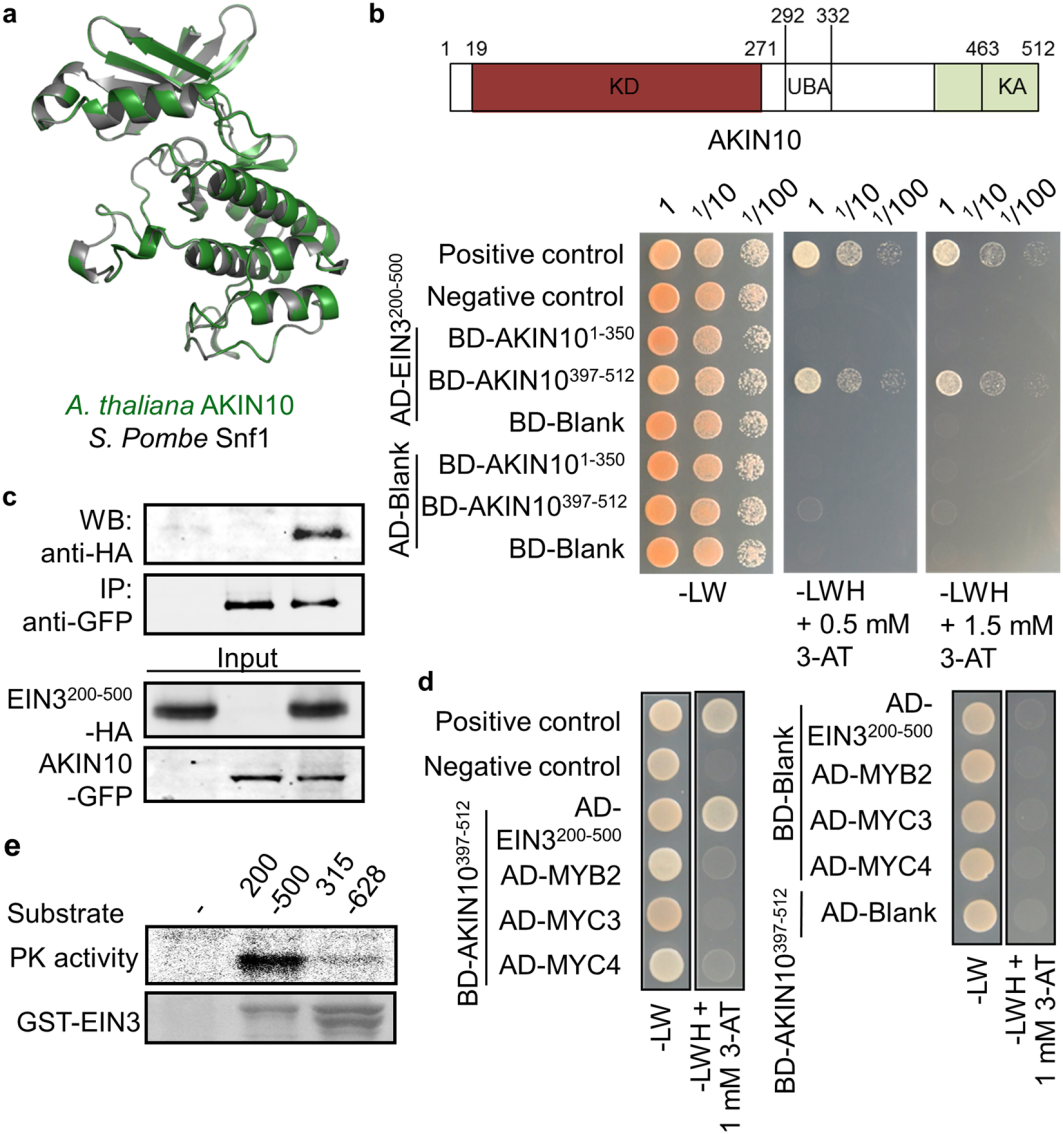
AKIN10 interacts directly with EIN3. (**a**) Structure modeling of protein kinase domain of Snf1 in *S*. *pombe* and AKIN10 in *A*. *thaliana*. Individual protein structures were generated using SWISS-MODEL (http://swissmodel.expasy.org) and visualized in a superimposed image using PyMOL (http://www.pymol.org). (**b**) Binary protein-protein interaction of AKIN10 and EIN3 was analyzed using a yeast two-hybrid system. (**c**) Protein-protein interaction of AKIN10 and EIN3 was confirmed by co-immunoprecipitation using *Arabidopsis* protoplasts transfected with a combination of *AKIN10* and *EIN3* constructs. (**d**) Binary protein-protein interactions between AKIN10 with MYB2, MYC3, or MYC4 were analyzed using a yeast two-hybrid system. (**e**) AKIN10-dependent EIN3 phosphorylation *in vitro* was shown with GST-EIN3 fragments as substrate. Coomassie blue staining was used for protein substrate visualization. All experiments were repeated with consistent results.

Signaling protein kinases from evolutionarily distant origins share some levels of similarity in their molecular structure. Protein functional orthologues carry even higher structural similarities in their kinase domains. Along with this notion our protein structure modeling revealed a highly conserved molecular conformation around catalytic domains of Snf1 in *Saccharomyces pombe* and AKIN10 in *Arabidopsis thaliana* (Fig. 1a), insinuating their essential and evolutionarily conserved cellular function.

In our small scale interaction screen of AKIN10 with several transcription factors involved in stress and hormone signaling such as MYB2, MYC2, MYC3, MYC4, JAZ and EIN3, we found AKIN10 interacted with MYC2 in abscisic acid and jasmonate, and EIN3 in ethylene signaling. We reported the AKIN10-MYC2 pathway for salt water tolerance in *Arabidopsis* ^25^. Here we closely assessed the regulatory function of AKIN10-EIN3 with respect to dark-inducible leaf senescence.

First, protein-protein interaction between AKIN10 and EIN3 was examined using a yeast-two-hybrid assay system. Because both full-length proteins of AKIN10 and EIN3 harbored auto-activation in the yeast strain used in the assay, various AKIN10 and EIN3 domains including AKIN10^1–350^, AKIN10^397–512^, EIN3^200–500^ and EIN3^315–628^ that do not carry auto-activities were identified and subjected for protein-protein interaction assay. The AKIN10^397–512^ and EIN3^200–500^ domains could overcome the innate metabolic deficiency of the yeast stain on the selection medium (-LWH) supplemented with 0.5 mM or 1.5 mM 3-AT (Figs 1b and S1). However, AKIN10^397–512^ could not do so with EIN3^315–628^, specifying the interaction between AKIN10^397–512^ and EIN3^200–314^.

To provide more evidence supporting this novel protein-protein domain interaction between AKIN10 and EIN3, co-immunoprecipitation was carried out with transiently expressed GFP-tagged AKIN10 and HA-tagged EIN3 in *Arabidopsis* protoplasts. Since the full-length EIN3 degrades rapidly in plant cells in the absence of ethylene^13, 14, 26^, co-immunoprecipitation was carried out with a stable EIN3^200–500^ domain instead of full-length protein. EIN3^200–500^ was successfully immunoprecipitated together with AKIN10 (Fig. 1c), confirming their binary interaction (Fig. 1b). In addition, AKIN10^397–512^ did not interact with other transcription factors such as MYB2, MYC3, or MYC4 in our yeast-two-hybrid assay (Fig. 1d), underlining the specific interaction of AKIN10 to EIN3.

We then examined whether AKIN10 could phosphorylate EIN3 through its direct interaction. A kinase assay *in vitro* was carried out with AKIN10 immuno-purified from protoplasts and GST-EIN3^200–500^ or GST-EIN3^315–628^ affinity-purified from bacterial cells as substrates. EIN3^315–628^ that did not interact with AKIN10 in the yeast-two-hybrid analysis served as a control substrate (Fig. S1). AKIN10 was able to phosphorylate only GST-EIN3^200–500^, not GST-EIN3^315–628^ (Fig. 1e), verifying AKIN10 phosphorylates EIN3 through direct interaction.

**Figure 2:**
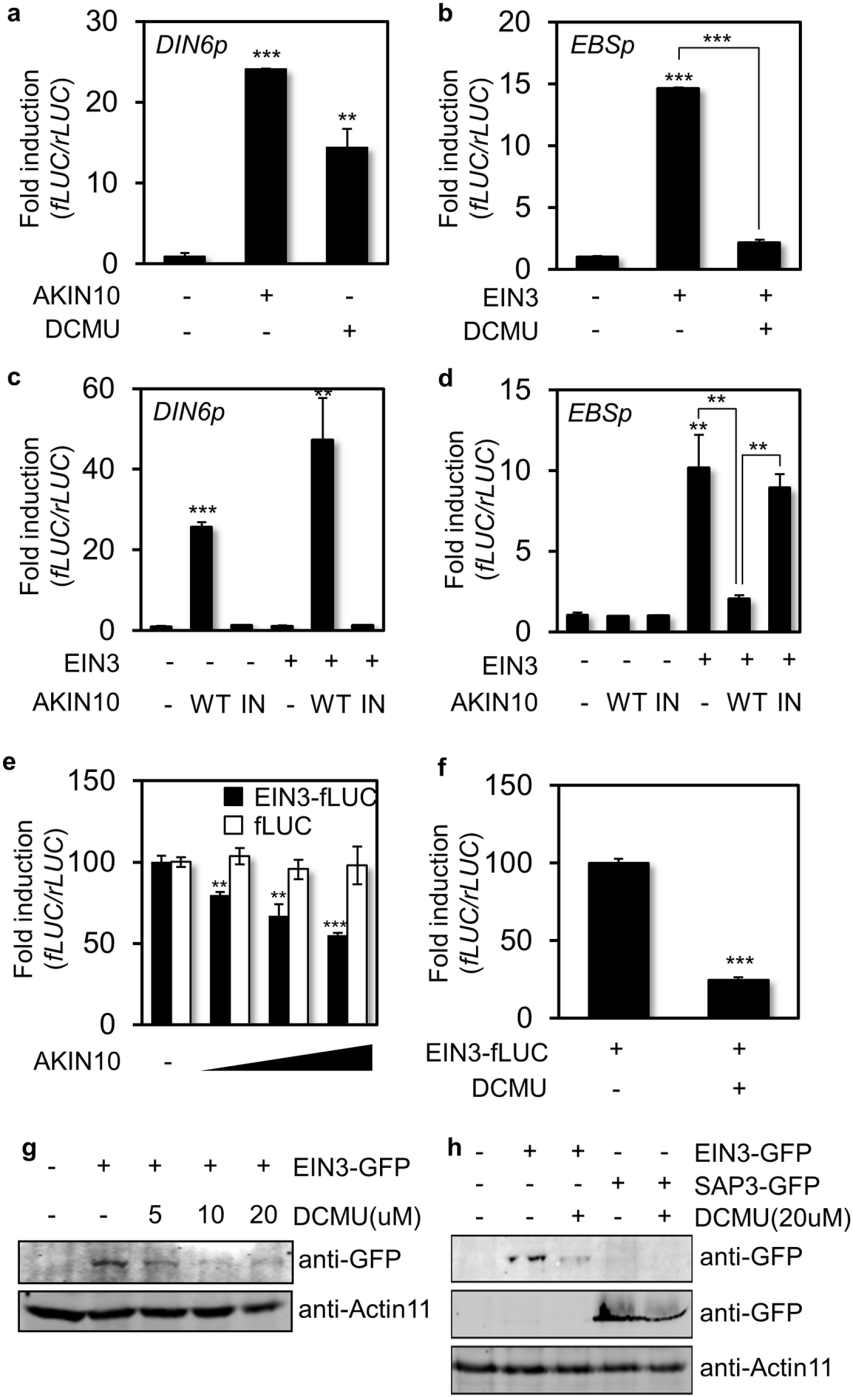
DCMU-inducible AKIN10 represses EIN3-dependent gene expression. (**a**,**b**) *DIN6* promoter (*DIN6p*) and *EBS* element (*EBSp*) activities were measured with expression of AKIN10 (**a**) or EIN3 (**b**) as well as in the presence of DCMU. (**c**,**d**) *DIN6p* (**c**) and *EBSp* (**d**) activities were measured in combination of active or inactive forms of AKIN10 with and without EIN3. (**e**) EIN3 protein level was measured as EIN3-fLUC activity with gradient expression of AKIN10 in *ein3-1* protoplasts. The fLUC activity was used as a cellular response control. (**f**) EIN3 protein level was measured as EIN3-fLUC activity in the presence of DCMU in *ein3-1* protoplasts. The rLUC activity served as control. All protoplast experiments were repeated three times with consistent results. The means of at least three replicates are shown with standard-error bars. ***P < 0.001, **P < 0.01, and *P < 0.05. (**g**,**h**) Protein levels of EIN3-GFP (**g**) and SAP3-GFP (**h**) were determined in *Arabidopsis* protoplasts in the absence and presence of DCMU by protein blot analysis using anti-GFP antibody. All experiments were repeated with consistent results.

To study the regulatory functions of AKIN10 on EIN3 actions, we established a cell-based functional assay with firefly luciferase (fLUC) reporters driven by AKIN10-inducible *DARK-INDUCED6* promoter (DIN6p)^21^ and EIN3-inducible *EIN3-binding-site* element (*EBSp*)^27^ using *Arabidopsis* protoplasts. DIN6p-fLUC activity was induced by AKIN10 expression as well as by DCMU treatment, a chemical elicitor of AKIN10 activity (Fig. 2a). EBSp-fLUC reporter activity was induced by EIN3 expression, but the EIN3-dependent induction of EBSp-fLUC activity was suppressed in the presence of DCMU (Fig. 2b), indicating DCMU enables to repress EIN3-dependent gene expression.

As previously reported^21, 24^, DIN6p-fLUC reporter activity was induced by wild-type (WT) AKIN10 (AKIN10^WT^), but not by the kinase inactive form of AKIN10 (AKIN10^IN^) that replaces Lys48 with Met. Moreover AKIN10-inducible DIN6p-fLUC activity was a little further enhanced by EIN3 co-expression, even though it was not induced by EIN3 alone (Fig. 2c). EBSp-fLUC reporter activity was not altered by either AKIN10^WT^ or AKIN10^IN^ alone and only induced by EIN3 expression (Fig. 2d). However, the induction of EIN3-dependent reporter activity was diminished by AKIN10^WT^ co-expression (Fig. 2d) in a manner similar to DCMU (Fig. 2b), but not by AKIN10^IN^. These results suggested that AKIN10 activation leads to the suppression of EIN3-dependent gene expression.

To understand how AKIN10 suppresses EIN3-inducible reporter activity, EIN3 protein stability regulation was examined as a putative functional mode of AKIN10. The short-lived EIN3 protein level was monitored quantitatively with a protein translation reporter generated by the translational fusion of *EIN3* cDNA to the N-terminal of *fLUC* gene driven by a constitutive *35* *S* promoter. EIN3 protein level was thus read out as fLUC activity when EIN3-fLUC was transiently expressed in *ein3-1* protoplasts. The reporter activity was decreased by AKIN10^WT^ expression in a dose-dependent manner (Fig. 2e) and also repressed in the presence of an AKIN10 inducer DCMU (Fig. 2f). On the contrary, an inactive form of AKIN10 did not affected EIN3-fLUC activity (Fig. S2), supporting the kinase activity of AKIN10 modulates EIN3 protein accumulation. To substantiate this result, a *35* *S* promoter-driven *fLUC* construct was used as a control in this assay. Unlike EIN3-fLUC, the control fLUC activity was not affected by AKIN10 expression (Fig. 2e). These were in agreement with protein accumulation patterns of EIN3-GFP in *Arabidopsis* protoplasts discerned by a protein blot analysis (Fig. 2g). Clearly the EIN3-GFP level was diminished by DCMU in a dose-dependent manner. On the contrary, an irrelevant protein SAP3 (STRESS ASSOCIATED PROTEIN3)-GFP level was maintained more or less after the treatment of DCMU, although DCMU-inducible AKIN10 activity influenced general translational processes and reduced SAP3 expression to some extent (Fig. 2h). Altogether, AKIN10 interacted with EIN3 in a binary protein interaction mode and its kinase activity compromised EIN3 protein accumulation in *Arabidopsis* cells.

### AKIN10 Controls EIN3 Accumulation and Suppresses Its Target Gene Expression

**Figure 3:**
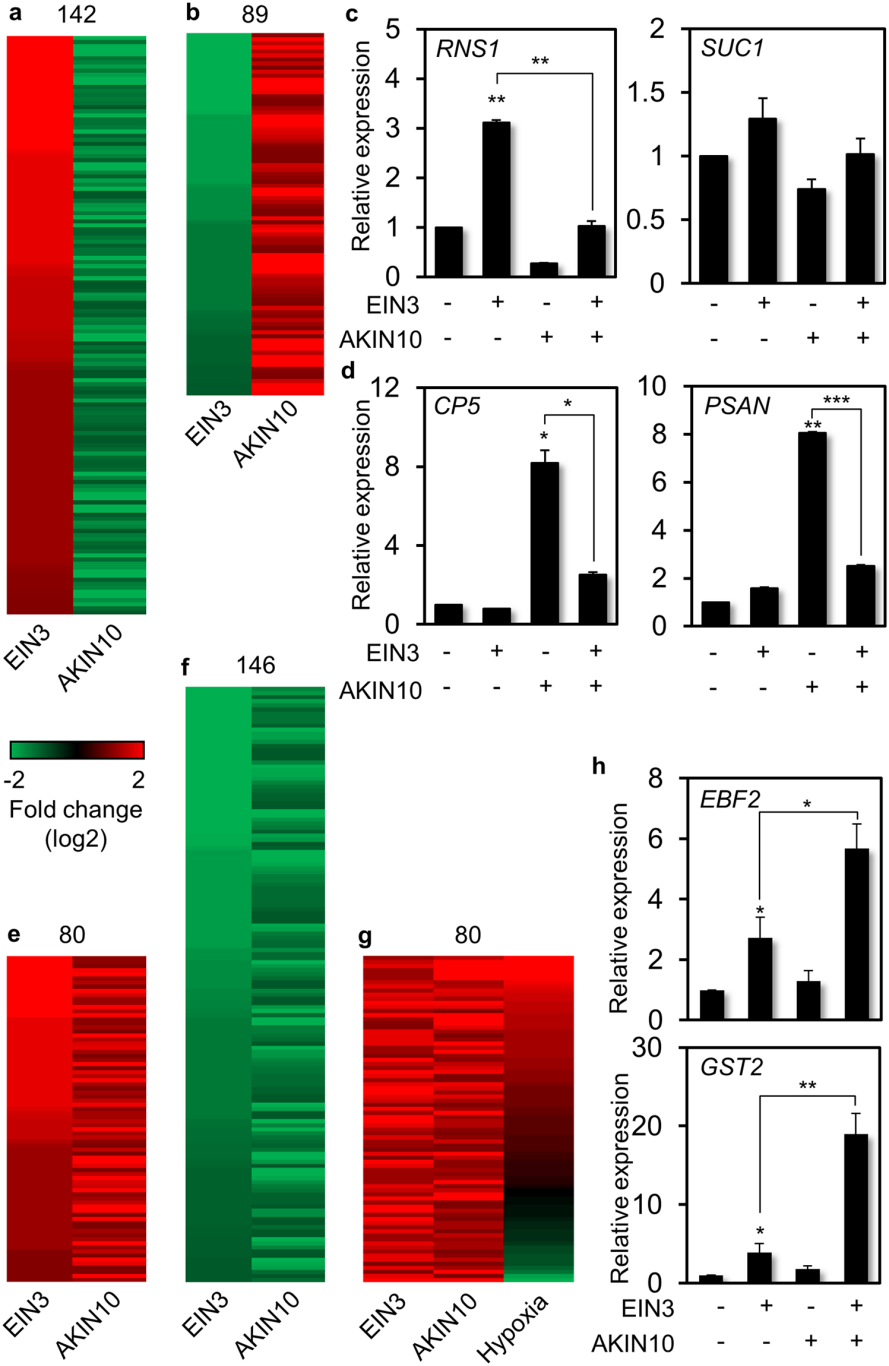
RNA sequencing-based analysis of differentially expressed genes (DEG) by EIN3 and AKIN10. (**a**,**b**) Comparative analysis of DEG up-regulated by EIN3 and down-regulated by AKIN10 (**a**) and those down-regulated by EIN3 and up-regulated by AKIN10 (**b**). (**c**,**d**) Expression of genes up-regulated by EIN3 and down-regulated by AKIN10 (**c**) and those down-regulated by EIN3 and up-regulated by AKIN10 (**d**) were measured using *Arabidopsis* protoplasts in combination of EIN3 and AKIN10 using RT-qPCR. (**e**,**f**) Comparative analysis of DEG up-regulated by both EIN3 and AKIN10 (**e**) and those down-regulated by both EIN3 and AKIN10 (**f**). (**g**) Meta-analysis of DEG up-regulated by both EIN3 and AKIN10 with those by hypoxia. (**h**) Expression of genes up-regulated by both EIN3 and AKIN10 was measured in combination of EIN3 and AKIN10 using RT-qPCR. (**c**,**d**,**h**) Values are means of triplicates with standard error bars (*p < 0.001; **p < 0.01; ***p < 0.05). All experiments were repeated with consistent results.

To further examine gene regulation by AKIN10 and EIN3 at a global genome scale, RNA sequencing (RNA-seq) was carried out with poly(A)-RNA isolated from *Arabidopsis* protoplasts transfected with *EIN3* and incubated for 6 h. Poly(A)-RNA was also isolated and sequenced from those transfected with the N-terminal half of *YFP*, gene products of which do not affect cellular processes, but still balance translational demands of the effector gene. The RNA-seq data were processed and analyzed to identify more than two-fold differentially expressed genes (DEG) upon EIN3 expression (CLC Genomics Workbench 10.0). We acquired 918 up-regulated and 1125 down-regulated genes by EIN3 in this analysis and these were compared with AKIN10 DEG data retrieved from public database, which was obtained from protoplasts transfected with AKIN10, incubated for 6 h, and analyzed by *Arabidopsis* ATH1 genome array analysis^21^. About a quarter of EIN3 DEG was inversely regulated by AKIN10 that is 142 DEG up-regulated by EIN3 was down-regulated by AKIN10 (Fig. 3a), and 89 DEG down-regulated by EIN3 was up-regulated by AKIN10 (Fig. 3b). We validated RNA-seq results with gene expression analysis of cherry-picked DEG using reverse transcriptase-dependent quantitative real-time PCR (RT-qPCR). *RIBONUCLEASE1* (*RNS1*) and *SUCROSE TRANSPORTER1* (*SUC1*) expression was induced by EIN3, but not by AKIN10 (Fig. 3c). Moreover, their EIN3-dependent induction was repressed by AKIN10. In contrast, polyketide cyclase/dehydrase-like *CP5* and photosystem I-associated *PSAN* expression was not induced by EIN3, but by AKIN10, and their AKIN10-dependent gene induction was repressed by EIN3 (Fig. 3d).

Our RNA-seq data analysis further identified 80 DEG up-regulated by both EIN3 and AKIN10 (Fig. 3e), and 146 of those down-regulated by both EIN3 and AKIN10 (Fig. 3f). Since both ethylene signaling and AKIN10 activity are reported to have a role in flooding-induced hypoxia tolerance in plants^24, 28, 29^, we examined whether DEG commonly regulated by EIN3 and AKIN10 was involved in hypoxia signaling response. To do so, DEG up-regulated by both EIN3 and AKIN10 were compared with DEG up-regulated by hypoxia retrieved from public database. Majority of the DEG co-regulated by EIN3 and AKIN10 was found in DEG up-regulated by hypoxia (Fig. 3g), supporting the notion that these DEG co-regulated by EIN3 and AKIN10 have a major role in hypoxia response. To verify gene expression responses that are up-regulated by both EIN3 and AKIN10, expression of *EIN3-BINDING F-BOX PROTEIN2* (*EBF2*) and *GLUTATHIONE-S-TRANSFERASE2* (*GST2*) was monitored using RT-qPCR. Indeed these genes were induced by EIN3 and further enhanced by co-expression of AKIN10, although their gene expression induction by AKIN10 alone was marginal (Fig. 3h). In summary a significant portion of EIN3 dependent gene expression is antagonistically modulated by AKIN10 possibly through their protein interaction and modification. Furthermore, our RNA-seq experiments were able to acquire meaningful gene expression patterns for multiple physiological outputs including hypoxia response under the regulation of two signaling components EIN3 and AKIN10.

### An AKIN10 elicitor DCMU Delays *Arabidopsis* Leaf Senescence

**Figure 4:**
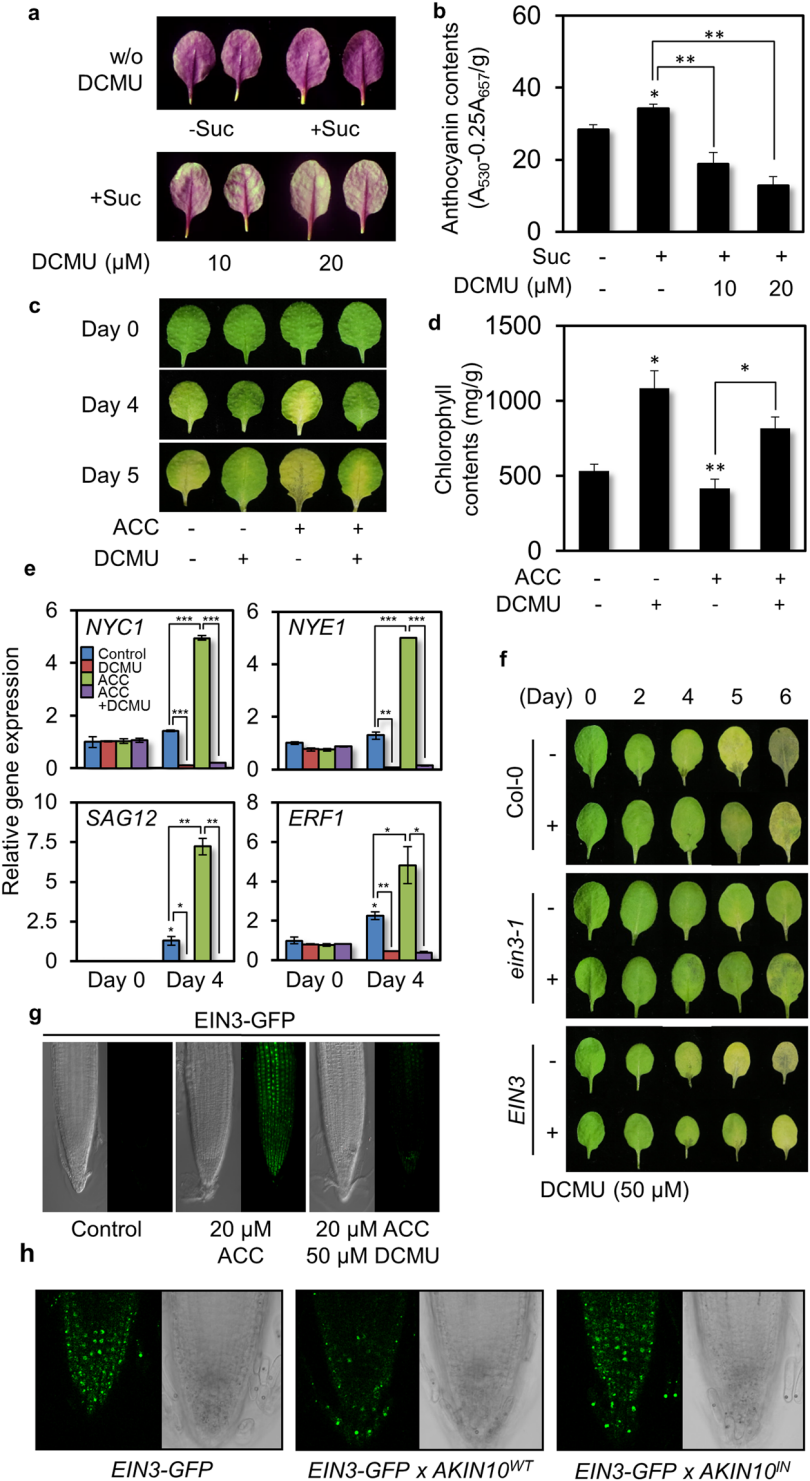
An AKIN10 elicitor DCMU delays EIN3-dependent ethylene inducible leaf senescence. (**a**,**b**) Anthocyanin accumulation was observed (**a**) and measured (**b**) in detached mature leaves in combination of sucrose and DCMU under light (n = 25). (**c**,**d**) Leaf degreening (**c**) was monitored and chlorophyll contents (**d**) were measured with detached mature leaves in combination of ACC and DCMU under darkness (n = 25). Values are means of triplicates with standard error bars (*p < 0.001; **p < 0.01; ***p < 0.05). (**e**) Expression of marker genes for senescence and ethylene signaling was monitored using quantitative RT-qPCR. *EIF4a* served as a control. (**f**) Leaf degreening was observed for Col-0, ethylene insensitive *ein3-1* and ethylene oversensitive *EIN3-*expressing transgenic lines (*EIN3*) in the absence and presence of DCMU. (**g**) Fluorescence signals were observed in root tips of *EIN3-GFP* expressing transgenic Col-0 in the presence of ACC and/or DCMU under confocal microscopy. (**h**) Fluorescence signals were observed in root tips of transgenic lines expressing *EIN3-GFP* and *AKIN10* ^*WT*^ or *AKIN10* ^*IN*^ in the presence of ACC under confocal microscopy. All experiments were repeated with consistent results. Representative results were shown.

We then observed leaf senescence progression in combination of AKIN10 elicitor DCMU and ethylene precursor 1-aminocyclopropane-1-carboxylic acid (ACC). In this study detached leaf senescence was monitored with the benefit of rapid and synchronous progression. Since individual leaves of a plant age differently, only the third and the fourth leaves of a fully expanded plant were used. We first examined leaf senescence in the presence of light. The color of detached leaves of Col-0 exposed to light became purple around the 4th day (Fig. 4a). The purple coloration reflects anthocyanin synthesis and accumulation as well as chlorophyll degradation driven by photosynthetic sugar production and accumulation^30^. Along with this notion the purple color got darker in the presence of 2% sucrose (Fig. 4a,b). Such coloration was then dramatically reduced in the presence of DCMU, implicating that AKIN10 activation by DCMU may slow down anthocyanin biosynthesis and chlorophyll degradation, indicators of plant senescence.

Detached leaves of Col-0 incubated in the dark became yellowish as they underwent chlorophyll degradation around the 4th day, which was further progressed at the 5th day (Fig. 4c). The dark-inducible leaf senescence was clearly slowed down in the presence of DCMU, but enhanced in the presence of ACC as ethylene promotes senescence progression^19^. Chlorophyll contents were relatively lower in Col-0 at the 4th day in the dark, but remained high in the presence of DCMU regardless of ACC (Fig. 4c,d). All results indicated together that DCMU delays leaf senescence, interestingly, independent of light conditions.

To examine gene expression responses to ACC/ethylene and/or DCMU during dark-inducible senescence, expression of marker genes related to ethylene responses (*ETHYLENE RESPONSE FACTOR1*) and senescence progression (*NON-YELLOW COLORING1*, *NON-YELLOWING1*, *SENESCENCE-ASSOCIATED GENE12*) was monitored using quantitative RT-PCR (RT-qPCR). Expression of *NYC1* (*NON-YELLOW COLORING1*) and *NYE1* (*NON-YELLOWING1*) coding for enzymes involved in chlorophyll degradation, and senescence-associated *SAG12* and ethylene-inducible *ERF1* (*ETHYLENE RESPONSE FACTOR1*) was detected at basal levels in detached leaves before dark incubation. These were induced during dark incubation and induced dramatically in the presence of ACC. On the other hand, such induction of these genes was clearly diminished in the presence of DCMU. These results indicated that DCMU induces delay of plant organ senescence, which is expedited by ACC/ethylene.

Since EIN3 is the transcription factor under AKIN10 regulation (Figs 2d and 3c), we investigated whether EIN3 modulation plays a regulatory role in the DCMU-induced delay of leaf senescence. Unfailingly leaf degreening due to chlorophyll degradation progressed in Col-0 during dark incubation (Fig. 4f). However, its progression was delayed in loss-of-function *ein3-1* and enhanced in *EIN3* expressing transgenic Col-0 (*EIN3*). In the presence of DCMU, leaf degreening progression was slowed down in both Col-0 and *EIN3-*expressing transgenic Col-0. However, the effect of DCMU on leaf degreening was not so obvious in *ein3-1*. Consistently, chlorophyll contents were relatively low in Col-0 after 4 days of dark incubation, and even lower in *EIN3* expressing transgenic line, but they remained high in *ein3-1* (Fig. S3). In the presence of DCMU, chlorophyll contents were maintained to a high level in Col-0 and *EIN3* expressing transgenic Col-0, but those were not affected in *ein3-1*. In summary, DCMU was able to modulate leaf senescence through EIN3 regulation in ethylene signaling.

To examine the notion that DCMU acts through EIN3 regulation in plants, transgenic Col-0 expressing EIN3-GFP was generated and selected for single inserted homozygous lines (Fig. S4a) and subjected to monitor its protein accumulation in response to DCMU. Transgenic seedlings were grown on an half-strength MS agar plates supplemented with 0.5% sucrose for 5 days and transferred to MS medium containing ACC (20 μM) and/or DCMU (50 μM). EIN3-GFP signals were observed in the presence of ACC, but not in the absence of ACC (Fig. 4g). The ACC-induced fluorescence signal was drastically reduced in the presence DCMU. In turn EIN3 protein stability was compromised by DCMU as shown in plant cells (Fig. 2g). Likewise, *Arabidopsis* root hair induction that is known to be accelerated in the presence of ACC/ethylene^31–33^ was blocked by DCMU as well (Fig. S4b). To further verify the notion that EIN3 is under the negative regulation by DCMU that is a potent inducer of AKIN10, we generated double transgenic plants expression *EIN3-GFP* and *AKIN10* ^*WT*^ or *AKIN10* ^*IN*^ and selected lines with high levels of both transgenes (Fig. S4c). EIN3-GFP was detected in the presence of ACC (Fig. 4h). The fluorescence signal decreased in transgenic lines expressing *EIN3-GFP* and *AKIN10* ^*WT*^, but not in those expressing *EIN3-GFP* and *AKIN10* ^*IN*^. All results indicated consistently that DCMU perturbs ethylene responses through EIN3 destabilization perhaps through its AKIN10 activation.

### AKIN10 Modulates Dark-Induced Leaf Senescence

**Figure 5:**
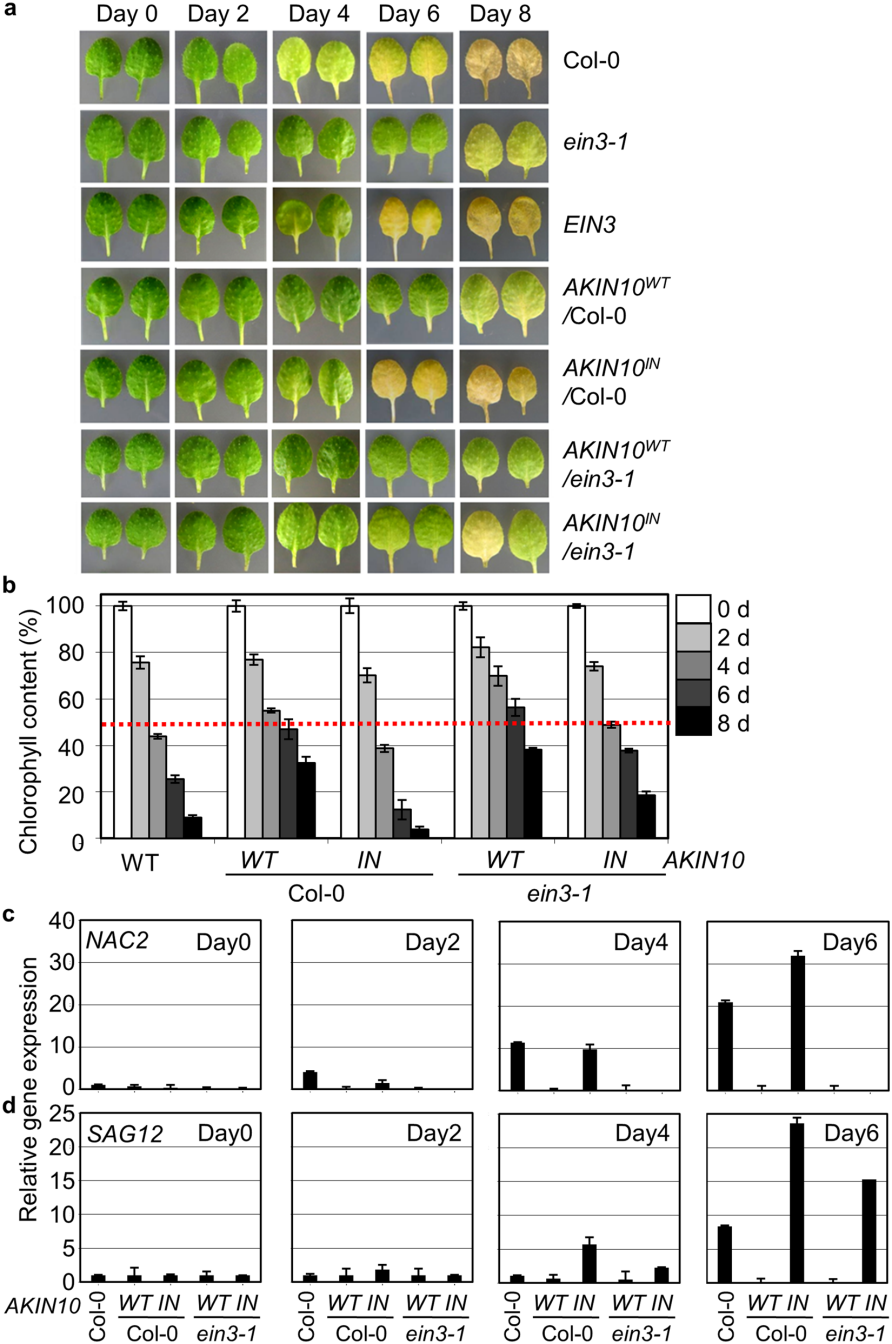
AKIN10 activity negatively modulates dark inducible leaf senescence. (**a**,**b**) Dark-inducible leaf degreening (**a**) was observed and chlorophyll contents (**b**) were measured for Col-0, *ein3-1*, transgenic Col-0 expressing *EIN3*, *AKIN10* ^*WT*^, or *AKIN10* ^*IN*^ and transgenic *ein3-1* expressing *AKIN10* ^*WT*^ or *AKIN10* ^*IN*^. Values were means of triplicates with standard errors (n = 5). Experiments were repeated with consistent results. (**c**,**d**) Expression of marker genes for early (*NAC2*) (**c**) and late (*SAG12*) (**d**) senescence responses was monitored using quantitative RT-qPCR. Values were means of triplicates with standard-error bars.

To genetically evaluate the antagonistic relationship between AKIN10 and EIN3-dependent ethylene signaling during leaf senescence, dark-inducible senescence was monitored with respect to AKIN10 activity in the presence or absence of EIN3 in whole plants (Fig. 5a). Transgene expression levels of transgenic plants were verified using semi-quantitative RT-PCR (Fig. S5a) and genetic make-ups of loss-of function mutants and transgenic lines were further confirmed using PCR-based dCAP analysis by allele-specific marker expression (Fig. S5b).

Detached leaves of *ein3-1* senesced slower, but those of *EIN3* expressing transgenic Col-0 plants senesced faster compared to Col-0 (Fig. 5a) as previously observed (Fig. 4f). Dark-inducible leaf senescence was progressed slowly in *AKIN10* ^*WT*^-expressing transgenic Col-0 plants, but rapidly in *AKIN10* ^*IN*^-expressing transgenic Col-0 plants in a manner similar to its natural senescence^24^. The senescence acceleration by AKIN10^IN^ was largely diminished in *AKIN10* ^*IN*^-expressing transgenic plants of *ein3-1* background. Chlorophyll contents were decreased drastically in detached leaves of *AKIN10* ^*IN*^-expressing Col-0 plants, but not as much in *AKIN10* ^*IN*^-expressing *ein3-1* (Fig. 5b) supporting the notion that AKIN10 modulates leaf senescence progression through EIN3 repression.

To monitor gene expression responses during leaf senescence, expression of senescence-related genes was monitored for an early response with *NAC2* (*NAC-DOMAIN CONTAINING PROTEIN*)^15^ and a late response with *SAG12* (ref. 18) using RT-qPCR. *NAC2* expression was induced in Col-0 at the 2nd day of dark incubation as an early senescence marker and was further increased as progressed (Fig. 5c). Gene induction was significantly reduced in detached leaves of *AKIN10* ^*WT*^-expressing transgenic Col-0 and transgenic *ein3-1* plants. Notably, *NAC2* expression was further enhanced in detached leaves of *AKIN10* ^*IN*^-expressing transgenic Col-0 at the 6th day, but not in those of *AKIN10* ^*IN*^ *-*expressing transgenic *ein3-1*. On the other hand, *SAG12* expression was induced in detached leaves of Col-0 at the 6th day, but it was induced earlier (at the 4th day) in those of *AKIN10* ^*IN*^-expressing transgenic Col-0 and further enhanced at the 6th day (Fig. 5d). Moreover, *SAG12* expression was largely reduced in *AKIN10* ^*WT*^-expressing transgenic Col-0 and transgenic *ein3-1* plants in a similar manner to *NAC2* expression. Unlike *NAC2*, *SAG12* expression was marginally reduced in *AKIN10* ^*IN*^-expressing transgenic *ein3-1*, implying that *SAG12* expression is not exclusively under the control of EIN3. All results consistently indicated that AKIN10 activity delays leaf senescence that is largely mediated through its antagonistic action on EIN3.

## Discussion

The present study enlightened the mechanistic pathway how an evolutionarily conserved cellular energy sensor AKIN10 activity establishes the calorie-restriction-inducible delay of organ senescence in a photosynthetic higher plant *Arabidopsis*.

Our cell-based functional and biochemical assays have shown that AKIN10 directly interacts with and phosphorylates EIN3 and thereby destabilizes the key transcription factor in plant senescence hormone ethylene signaling and down-regulates its target gene promoter activity (Figs 1 and 2). In the global gene expression analysis, a significant portion of subset of EIN3 inducible gene expression is antagonistically modulated by AKIN10 (Fig. 3). Consistently, senescence progression of detached leaves accompanying chlorophyll degradation in the absence and presence of light is expedited by ethylene/ACC, but slowed down by DCMU that induces AKIN10 protein kinase activation (Fig. 4). Taken together, when a senescence program is initiated in detached leaves, AKIN10 is activated to slow down its progression through EIN3 regulation as cellular energy deprives.

This notion could be examined with null mutants of two redundant protein kinases *akin10 akin11*. However, the double mutant has known to be seedling lethal and individual mutants have no obvious senescence phenotype^21^. To overcome such limitation in genetic analysis, we monitored detached leaf senescence of transgenic Col-0 expressed with a dominant negative *AKIN10* ^*IN*^ (K48M) and also employed *ein3-1* mutant background to dissect relation between AKIN10 and EIN3 in this study. Genetic interaction analysis between AKIN10 and EIN3 has clearly demonstrated that AKIN10 delays detached leaf senescence through EIN3 regulation (Fig. 5). However, EIN3 is not the only target of AKIN10 in organ senescence regulation as senescence-related *SAG12* expression increases in *AKIN10* ^*IN*^ expressing transgenic plants in *ein3-1* background. Perhaps other cellular pathways involved in senescence modulation such as autophagy could be under the regulation of AKIN10 activity in plants in a manner similar to those in yeasts and mammals^34^.

The AKIN10-inducible senescence delay demonstrated with detached leaves in this study has been previously reported for natural senescence of leaves as well^21, 24^. Such anti-senescence/aging action of AKIN10 is an evolutionarily conserved feature of its functional homologs in *Caenorhabditis elegans* and *Drosophila melanogaster*, although specific regulatory paths have diverged from each species^35, 36^.

Antagonistic regulation of EIN3 by AKIN10 further explains how EIN3 becomes stable in the light, but unstable in the dark^37^. Rationally, the lack of photosynthetic activity of chloroplasts in darkness leads to cellular energy deprivation and thereby AKIN10 activation, in which EIN3 accumulation is seemingly repressed. In contrast, high photosynthetic activity under light suppresses AKIN10 activity, and provides cellular conditions for EIN3 to accumulate to a relatively higher level. In the same line of evidence, overexpression of *AKIN10* conferred some degree of ethylene insensitivity in etiolated seedlings in the presence of ethylene (Fig. S5c).

In the study our attempt to identify phosphorylation sites of EIN3 by AKIN10 activity using mass spectrometry was unfertile. The preliminary analysis of phospho-proteomics found more than three amino acid sites for phosphorylation within the AKIN10 interacting domain of EIN3. Further research will provide more detailed mechanistic information of AKIN10-dependent EIN3 regulation.

Here we showed that DCMU treatment slows down senescence-associated chlorophyll degradation in detached leaves in the absence and presence of light. DCMU function in the dark was previously reported with regards to cell cycle regulation in a photosynthetic unicellular organism *Euglena* ^38^. Furthermore, the regulatory mechanism of DCMU under light that blocks the early step of electron transport chain at PSII instructs that the more greening by DCMU treatment does not signify the more photosynthetically functional chloroplasts. Even so, because keeping green is a valuable asset for vegetable crops, it is worth noting that DCMU can control the degreening progression of vegetative organs in postharvest physiology. This information may allow plant industry to yield a strategy for postharvest management to maximize commodity values by extending shelf life in the marketplace.

## Materials and Methods

### Plant materials and growth conditions

*Arabidopsis* plants were grown in soil at 24 °C under photon irradiance of 60 µmol·m^−2^·s^−1^ and with a 12-h photoperiod. *Arabidopsis* Columbia-0 (Col-0) as the wild type and *ein3-1* were used for experiments. Plasmid constructs for transgenic plants were generated by inserting the cDNA of WT AKIN10 (AKIN10^WT^) or catalytically inactive ATP-binding site mutant AKIN10^K48M^ (AKIN10^IN^) between the *35SC4PPDK* promoter and the *NOS* terminator in a mini-binary vector, p*CB302* (ref. 24).

For leaf senescence assay, plants were grown in an environmentally controlled growth chamber at 24°C with a 16 h light and 8 h dark photoperiod at 80 µmol·m^−2^·s^−1^. *Arabidopsis* rosettes with fully developed leaves detached from three-week-old plants were placed on 9-mm-diameter petri dishes with triple-layer Whatman filter papers in the base containing 15 ml distilled water. Only the third and the fourth leaves of a given plant were used for further analyses. The petri dishes were placed in the dark at room temperature for designated time period. For chemical treatment, detached leaves were incubated in 3 mM MES solution with 100 µM ACC and/or 50 µM DCMU and harvested for further analyses. Alternatively, detached leaves were placed on 3 mM MES buffer containing 2% sucrose or DCMU with a 16 h light/8 h dark cycle in the absence or presence of light (80 µmol·m^−2^·s^−1^).

The ethylene sensitivity assay was performed on etiolated seedlings in complete darkness for three days with or without ACC (Sigma-Aldrich, St. Louis, MO, USA). After four days of stratification, seeds were germinated on MS agar plates (Caisson Biotech, Austin, TX, USA) supplemented with 1% (w/v) sucrose (Duchefa Biochemie, Haarlem, Netherlands) to ensure uniform germination.

### Chlorophyll and anthocyanin content measurements

Chlorophyll was extracted from individual leaves using 100% ethanol and the concentration per fresh weight of leaf tissue was calculated as described previously^24^.

To extract anthocyanin, leaves were frozen with liquid nitrogen and ground up to a fine powder. Five volumes of extraction buffer (45% methanol and 5% acetic acid) were added and incubated at 4 °C for 1 h. Cell debris was removed by two rounds of centrifugation. Absorbance at 530 and 657 nm was measured and anthocyanin contents were calculated as previously described^39^.

### Transient transfection of *Arabidopsis* mesophyll protoplasts

Protoplast transient transfection assays were performed using the polyethylene glycol-calcium transfection-mediated method described previously^40, 41^. Constructs for *AKIN10* ^*WT*^, *AKIN10* ^*IN*^, and *EIN3* were generated by inserting cDNA between the *35SC4PPDK* promoter and *NOS* terminator in a plant expression vector. Promoter regions of *EBSp* ^27^ and *DIN6p* (−624 to −1)^21^ were cloned into a firefly luciferase-conjugated plant expression vector. *UBQ10-RenillaLUC* (*UBQ10-rLUC*) was co-transfected as an internal transfection control for the reporter assay. To study the role of AKIN10 and/or EIN3, three plasmids expressing a regulatory effector(s), a specific reporter and a transfection control reporter were used at the ratio of 16:3:1. The same total plasmid DNA amount was used or was compensated with a control plasmid that did not express any effectors and incubated at room temperature for 7 h. Promoter activity was expressed as the firefly luciferase activity/renilla luciferase activity (fLUC/rLUC) ratio and normalized to the values obtained without treatment or effector expression.

### co-Immunoprecipitation and Protein immunoblotting

Total protein was extracted from the transfected protoplasts using immunoprecipitation (IP) buffer containing 50 mM Tris-HCl pH 7.5, 150 mM NaCl, 5 mM EDTA, 1 mM DTT, 1% Triton-X100, and Complete™ protease inhibitors (Roche, Basel, Switzerland). After incubation on ice for 10 min, the protein extract was centrifuged at 13000 rpm for 5 min at 4 °C to remove cell debris. For pre-clearing, 10 μL of Protein A-agarose (Roche) was added to the cleared protein extract, which was then incubated for 30 min at 4 °C. Protein A-agarose was separated by centrifugation for 1 min at 4 °C and supernatants were transferred into a new tube. The protein extract was incubated first with anti-GFP antibody (Clontech Laboratories) for 3 h and then with a Protein-A bead for 6 h at 4 °C. After washing three times with IP buffer (without Triton-X), the pellet was resuspended in SDS sample buffer. The extract was boiled at 95 °C for 10 min and centrifuged. The supernatants were loaded onto a 12% SDS-PAGE gel and transferred to a PVDF membrane (Millipore, Billerica, MA, USA). After blocking using 5% skim milk in Tris buffered saline-Tween20, the membrane was incubated first with anti-HA (Roche) (1:1,000) or anti-GFP (1:1,000) antibodies and then with infrared-800-conjugated anti-mouse antibody (Li-Cor) (1:10,000). The signal was detected using a Li-Cor IR-image scanner (Odyssey). The results were confirmed with at least two replications.

### Protein kinase assay *in vitro*

Substrates for the protein kinase assay were prepared from the transfected *Escherichia coli* cells. Expression of GST-EIN3^200–500^ and GST-EIN3^315–628^ was induced at 37 °C for 3 h in the presence of 2 mM IPTG (USB) and harvested by centrifugation at 4200 rpm for 20 min. The cell pellet was resuspended in 1 mL PBS and physically lysed by sonication (Bioruptor; Diagenode). The cell lysate was incubated in PBS with Complete™ protease inhibitor and 1% Triton-X for 30 min at 4 °C. Cell debris was removed by centrifugation at 13000 rpm for 5 min and the supernatant was incubated with glutathione (GST) agarose (Thermo Scientific, Pittsburgh, PA, USA) at 4 °C for 1 h. The GST agarose was washed three times with washing buffer (50 mM Tris-HCl, pH 8.0 and 150 mM NaCl) and the GST fusion protein was eluted with elution buffer (50 mM Tris-HCl pH 8.0, 150 mM NaCl, and 15 mM reduced Glutathione).

Protein kinase was expressed and immuno-purified from *Arabidopsis* protoplasts transfected with *AKIN10*. Total proteins were extracted from protoplasts using kinase buffer containing 20 mM Tris-HCl pH 7.5, 40 mM MgCl_2_, 5 mM EDTA, 1 mM DTT, 10 mM NaF, 10 mM Na_3_CO_4_, 1% Triton-X, and Complete™ protease inhibitor. After centrifuging to removing cell debris, Protein A-agarose was added and the reaction mixture was incubated for 30 min for pre-clearing. The supernatant was incubated first with anti-GFP antibody for 3 h and then with Protein A-agarose for 3 h. The beads were washed once with IP buffer (without Triton-X) and twice with kinase buffer (without Triton-X). The pellet was resuspended in kinase reaction buffer (25 mM Tris-HCl pH 7.5, 10 mM MgCl_2_, 1 mM EGTA, 1 mM DTT, 0.2 mM ATP, and 1.5 μL Ci γ-^32^P ATP) and incubated with GST-EIN3^200–500^ or GST-EIN3^315–628^ at 30 °C for 30 min. After the reaction was complete, the mixtures were mixed with SDS sample buffer and resolved by12% SDS-PAGE. After washing five times with water, radioactivity was detected using a radioactivity detector (PMI, Bio-Rad).

### Protein binary interaction assay

Yeast two-hybrid assays were performed using the Matchmaker^TM^ GAL4 two-hybrid system (Clontech Laboratories, Mountain View, CA, USA) according to the manufacturer’s instructions. The EIN3 fragment^200–500^ was subcloned into the *pGADT7* vector for GAL4 AD, and the AKIN10^1–350^ and AKIN10^397–512^ fragments were subcloned into the *pGBKT7* vector for GAL4 BD. The constructs were transformed into the yeast strain *AH109* (-Leu, -Trp, -His, -Ade). Yeast transformants were grown on synthetic dropout (-Leu, -Trp, -His) medium plates containing either 0.5 mM or 1.5 mM 3-AT to confirm the interaction between the two proteins. Yeast cells were grown at 30 °C for 3 to 4 days.

### RNA isolation and transcript measurement

For gene expression analysis, total RNA was isolated using RNAiso Plus (Takara Bio, Shiga, Japan) from leaves, and 1 µg of total RNA was used for cDNA synthesis using Moloney murine leukemia virus reverse transcriptase (Promega, Madison, WI, USA). Gene expression was measured using real-time PCR (CFX Connect^TM^ Real-Time System with C1000 Thermal Cycler, Bio-Rad) and an iQ SYBR Green dye-supplemented PCR mix (Bio-Rad). Either *Tubulin4* (*TUB4*, At1g04820) or *Elongation initiation factor4a* (*EIF4a*, At3g13920) transcripts were used as a real-time PCR control with gene-specific primers. Primer sequences are presented in Supplemental Table S1. The production of a single gene product by each primer set in PCR reactions was validated before the experiment. Experiments were repeated three times with consistent results.

### RNA-sequencing

To prepare RNA-sequencing sample, leaf mesophyll protoplasts were individually transfected with *EIN3* and *AKIN10* constructs were and incubated for 6 h under dim light. Total RNA was extracted using RNAiso Plus and then poly(A)-RNA was further isolated using Dynabead mRNA DIRECT^TM^ Kit (Thermo Scientific). Library preparation and Nucleotide sequencing were conducted with the manufacture’s instruction with Ion PGM^TM^ (Thermo Scientific). Raw reads were normalized and analyzed using CLC Genomics Workbench (QIAGEN, Valencia, CA, USA) ver.10 platform. To identify differentially expressed genes (DEGs), gene expression levels were normalized with RPKM values, and then analyzed with the Baggerley’s test as a statistical analysis tool using duplicated RNA-sequencing data. Genes with more than 2-fold (p < 0.05) changes were presented selected as DEGs (Supplementary datasets) and expressed into heat maps.

## Electronic supplementary material

Supplementary Figures

Supplementary Dataset
