# Supplementary material for: Regulatory Functions of Cellular Energy Sensor SNF1-Related Kinase1 for Leaf Senescence Delay through ETHYLENE- INSENSITIVE3 Repression
Source: Sci Rep. 2017 Jun 9;7:3193. doi: 10.1038/s41598-017-03506-1 (PMC5466610; doi:10.1038/s41598-017-03506-1)

Regulatory Functions of Cellular Energy Sensor SNF1-Related Kinase1 for Leaf Senescence Delay through ETHYLENE- INSENSITIVE3 Repression

Geun-Don Kim[1,2] , Young-Hee Cho[1,2] and Sang-Dong Yoo[2*]

[2]Department of Life Science, College of Life Science and Biotechnology, KOREA University, Seoul, Korea 02841

Correspondence*:

Sang-Dong Yoo

Department of Life Science

College of Life Science and Biotechnology

KOREA University

145 Anamro, Seongbuk-gu

Seoul, Korea, 02841

Tel: +82-2-3290-3401

Fax: +82-2-927-9028

E-mail: sangdong@korea.ac.kr

Footnote: [1]Theses authors contribute equally to this study.

a

	AD	BD
1	Positive Control	
2	Negative Control	
3	EIN3$^{200\text{-}500}$	AKIN10
4	EIN3$^{200\text{-}500}$	AKIN10$^{1\text{-}292}$
5	EIN3$^{200\text{-}500}$	AKIN10$^{1\text{-}350}$
6	EIN3$^{200\text{-}500}$	AKIN10$^{1\text{-}396}$
7	EIN3$^{200\text{-}500}$	AKIN10$^{397\text{-}512}$
8	EIN3$^{200\text{-}500}$	Blank
9	EIN3$^{315\text{-}628}$	AKIN10$^{397\text{-}512}$
10	Blank	AKIN10$^{397\text{-}512}$

b

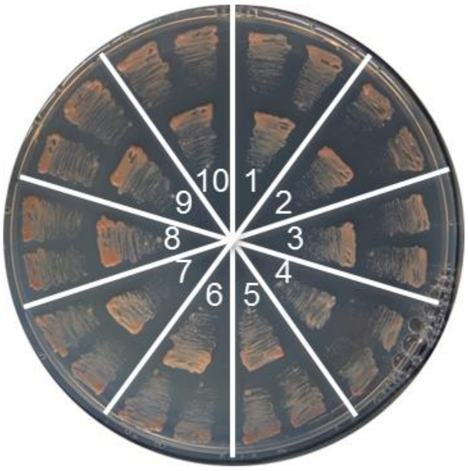

-LW

c

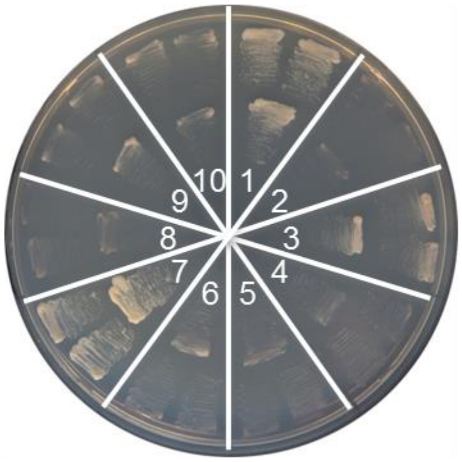

-LWH + 0.5 mM 3-AT

Supplementary Fig. S1. Binary interaction of AKIN10 with EIN3 in Y2H assay. (**a**) Table of interacting partners tested in Y2H assay. (**b**) Yeast growth phenotypes on transformation screen media (-LW). (**c**) Yeast growth phenotypes on interaction screen media (-LWH containing 0.5 mM 3-AT). Experiments were repeated with identical results. A representative set of results is shown.

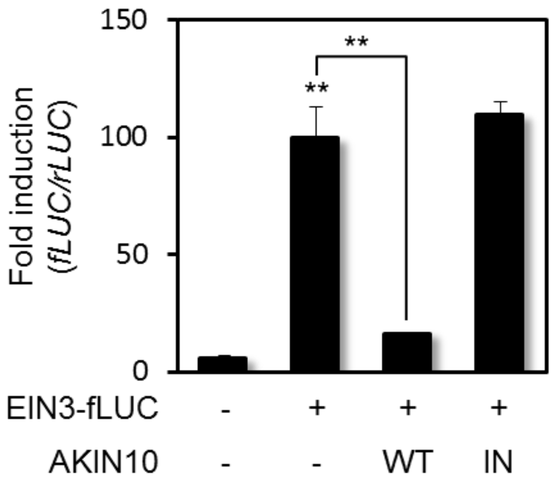

Supplementary Fig. S2. EIN3 protein level was measured as EIN3-fLUC activity in the absence and presence of AKIN10 in WT protoplasts. The rLUC activity served as control. All protoplast experiments were repeated three times with consistent results. The means of at least three replicates are shown with standard-error bars. ***P < 0.001, **P < 0.01, and *P < 0.05.

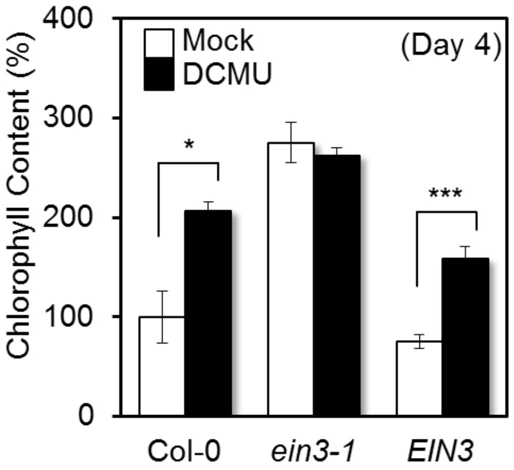

Supplementary Fig. S3. Chlorophyll contents were measure for Col-0, *ein3-1* and *EIN3* in the absence and presence of DCMU after dark incubation for 4 days. Values are means of triplicates with standard error bars ($p<0.001$; ***, $p<0.01$; **, $p<0.05$; *). All experiments were at least duplicated with consistent results.

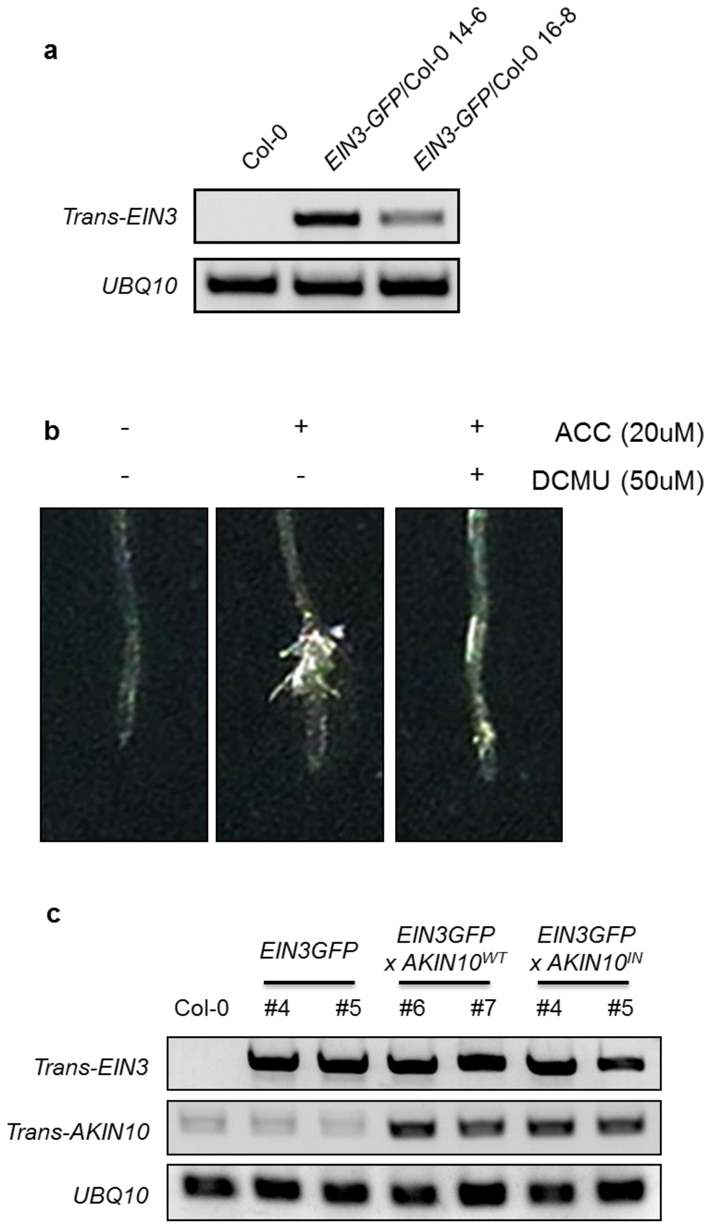

Supplementary Fig. S4. Transgene expression in transgenic Col-0 expressed with *EIN3-GFP*. (**a**) Gene expression was monitored using semi-quantitative RT-PCR. *UBQ10* served as a control. (**b**) *Arabidopsis* root hair induction in combination with ACC and DCMU. (c) Transgene expression levels were evaluated using semi-quantitative RT-PCR. *UBQ10* served as a control. EIN3-GFP/Col-0 and either L*er*, $AKIN10^{WT}$/L*er* or $AKIN10^{IN}$/L*er* were crossed and selected for double transgene expression lines. Experiments were run in triplicate with consistent results. A representative set of image was shown.

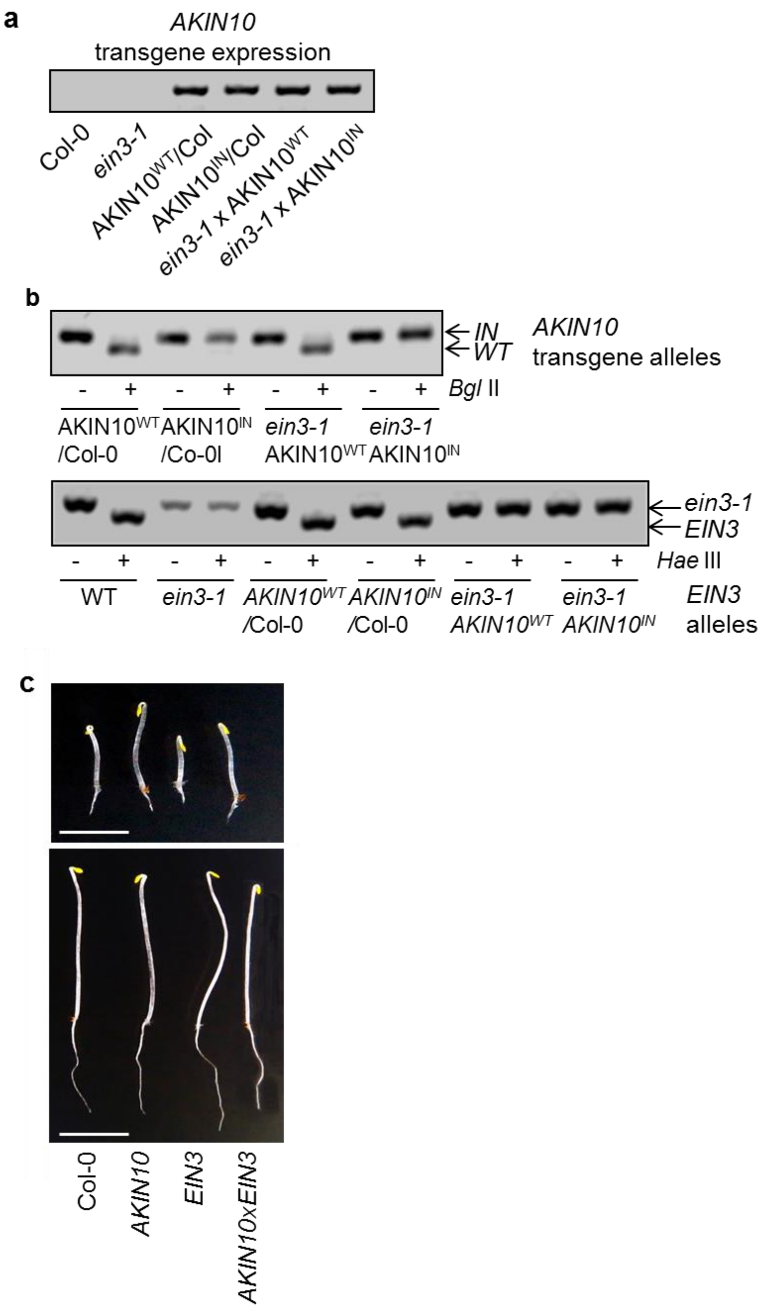

Supplementary Fig. S5. The validation of transgene expression in newly generated transgenic plants. (**a**) Transgene expression of transgenic Col-0 and transgenic *ein3-1* expressed with $AKIN10^{WT}$ or $AKIN10^{IN}$ were shown. (**b**) Allele-specific molecular markers and transgene expression of transgenic Col-0 and *ein3-1* expressed with $AKIN10^{WT}$ or $AKIN10^{IN}$ were shown. (**c**) Triple-response assay of Col-0, *AKIN10*-expressing transgenic Col-0, *EIN3*-expressing transgenic Col-0, and *AKIN10* and *EIN3*-expressing transgenic Col-0 seedlings. Scale bar, 5 mm. Experiments were repeated three times with consistent results.

Supplementary Table S1. Oligonucleotides used in this study

Oligo name	Oligonucleotide (5'-3')
**dCAPs PCR primers**	
AKIN10_f	GATTTTGCGACGATTGAAGATC (*Bgl*II/cut WT)
AKIN10_r	AGAATGGATGGATCAGGCAC
EIN3_f	GAGCAAGCTAGGAGGAAGAAATGTCTAG (*Hae*III/cut WT)
EIN3_r	TTTAGGCAAACCAAGTTGGATGCCAC
**Gene expression PCR primers**	
AKIN10_f1	ATGAAGTGCAGATGGGTTCC
AKIN10_r1	GCAGCACACAGATCCAAGAA
CP5_f	TATGGGAATACCGTGGGAGA
CP5_r	TGGTGTTGATGTGAGCCATT
CAB2_f	GCTAGAAGTTATCCACAGCAG
CAB2_r	CGACTCTGTAACCTTCAACG
EBF2_f	CGGCTGCTCCTCAATTACAG
EBF2_r	CAGTGCTGCTGCTGATTCTG
ERF1_f	AGTCGACAGCGAGTTCGGTTAC
ERF1_r	AGCTAGGGTTTCGTCCGTACAC
GST2_f	TCCAAACCGACTCCAAGAAC
GST2_r	GCCTCCTCTTCTGCAACAAC
NAC2_f	ACGTGCCGATGGTACAAAGGTTC
NAC2_r	TCTTGGTCGGAGAAGCAGGTCAC

NYC1_f	AAACAAACGTTGGCCTTCAC
NYC1_r	TTTCCCGAACCTTTCACAAC
NYE1_f	GCCTGATGGTCACAAGACTG
NYE1_r	CTCCGGATTTGGAGTAGCAA
PSAN_f	TCGAGAGGAGCAAAACCAAC
PSAN_r	ATCTTGGCAGCCAGTGAAAT
RNS1_f	TCTAACCAAAGCCGGGATTA
RNS1_r	TTCGATTAAACCGGAACCAG
SAG12_f	GCTGTGACCCCTATCAAG
SAG12_r	GATCCGTTAGTAGATTCGC
SUC1_f	CTCAAGGACATCCCCTGTGT
SUC1_r	CTCTCTGGCCAGTTTTACGC
TUB4_f	AGGGAAAGGAAGAGAGGAAG
TUB4_r	GCTGGCTAATCCTACCTTTGG
UBQ10_f	AGATCCAGGACAAGGAGGTATTC
UBQ10_r	CGCAGGACCAAGTGAAGAGTAG
ELF4a_f	TCATAGATCTGGTCCTTGAAAC
ELF4a_r	GGCAGTCTCTTCGTG

Figure 1 (c)

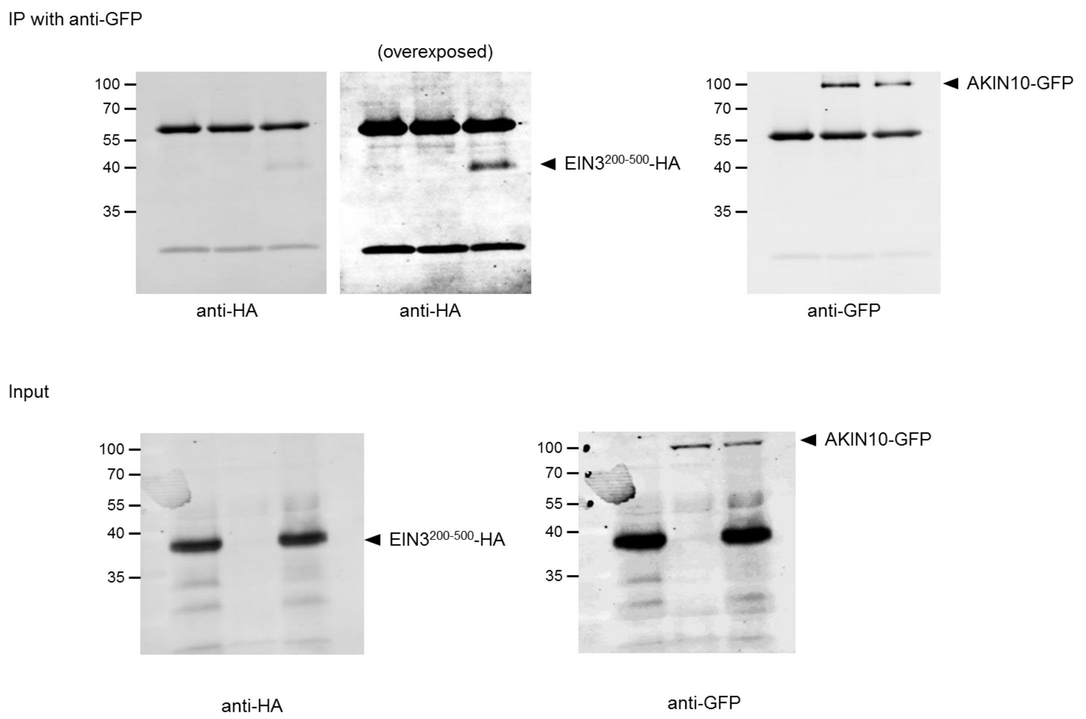

Figure 1 (e)

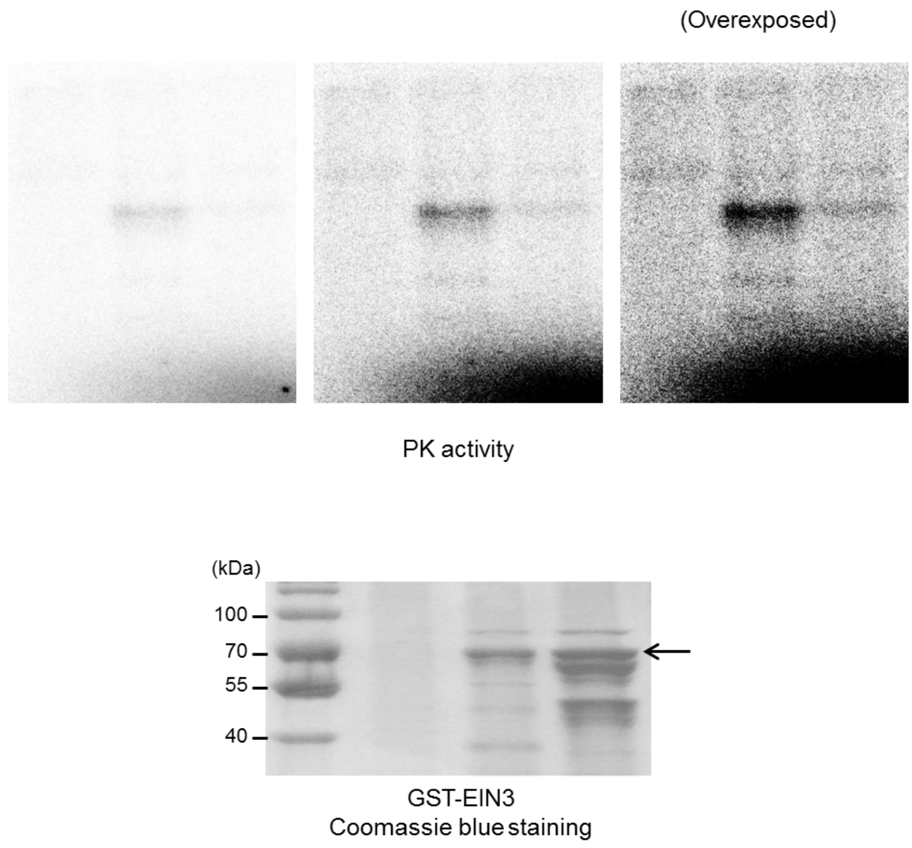

Figure 2 (f)

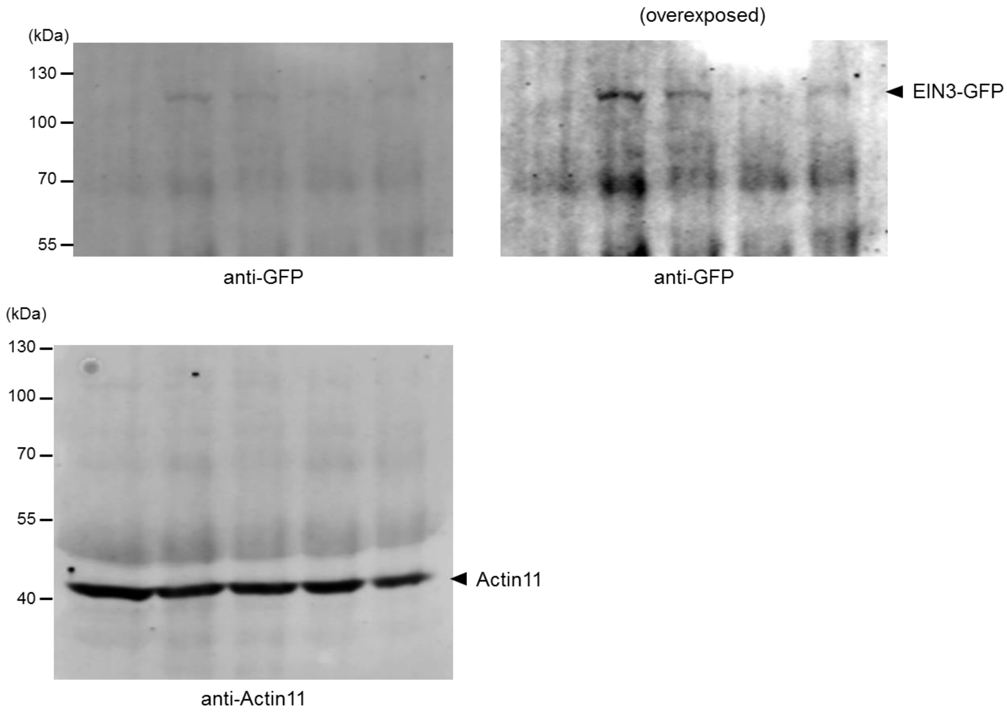

Figure 2 (g)

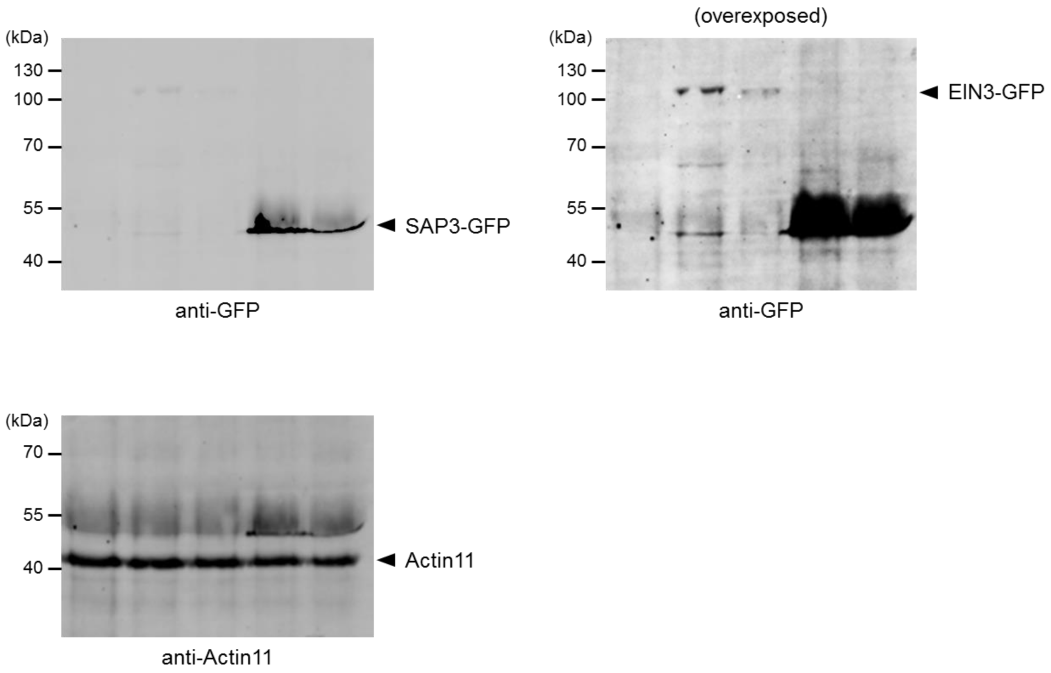

Supplement: Supplementary file 1 — Supplementary Figures [file 41598_2017_3506_MOESM1_ESM.pdf]
